# A rare case of a nasal cavity fungus ball due to *Aspergillus niger*

**DOI:** 10.18502/cmm.8.3.11213

**Published:** 2022-09

**Authors:** Emine Nur Kahraman, Şehrazat Evirgen, Ahmed Badri Abed, Safiye Elif Korcan, Cansu Gül Efeoğlu Koca

**Affiliations:** 1 Department of Oral and Maxillofacial Radiology, Faculty of Dentistry, Istinye University, Istanbul, Turkey; 2 Department of Oral and Maxillofacial Radiology, Faculty of Dentistry, Usak University, Usak, Turkey; 3 Department of Molecular Biology and Genetics, Faculty of Arts and Sciences, Uşak University Uşak, Turkey; 4 Department of Microbiology, Uşak Health Training School, Uşak University, Uşak, Turkey; 5 Department of Oral and Maxillofacial Surgery, Faculty of Dentistry, Usak University, Usak, Turkey

**Keywords:** Aspergillosis, *Aspergillus niger*, Fungus balls, Nasal cavity

## Abstract

**Background and Purpose:**

Fungus Ball (FB) is a non-invasive fungal infection caused mainly by *Aspergillus* species. It can occur after root canal treatments are applied to the teeth adjacent to the maxillary sinus. These balls are commonly seen in the paranasal sinuses and rarely observed in the nasal cavity. This report attempted in to highlight such a rare case of fungal infection which requires accurate observation. Moreover, it highlights the importance of careful microbiological and histopathological examinations that were combined with imaging and can lead to a definitive diagnosis.

**Case report:**

Herein, we report a rare case of a FB found in the vicinity of the nasal cavity of a 73-year-old male patient. Microbiological examination supported by radiographic and histopathological results indicated that the FB is due to *Aspergillus niger*. Excised surgery was done to the FB area, and the patient was referred to the post-operation room with the proper recommendations. After the wound healed, the total denture was performed as requested by the patient, and his overall oral health was improved.

**Conclusion:**

In this article, we report the first case of a rare FB in the vicinity of the nasal cavity of a 73-year-old male patient. The appropriate investigation is an essential step in the diagnostic process for these infections and requires effective communication and collaboration.

## Introduction

*Aspergillus* is a type of fungus with filaments that reproduce by spores, and very few of them are pathogenic for humans [ 1 ]. Infections of *Aspergillus* may present clinically in three different ways: allergic sinusitis, fungus ball, or invasive aspergillosis.

The Fungus Ball (FB) is classified as a non-invasive fungal infection caused mainly by the species *Aspergillus fumigatus* [ 2 , 3 ]. It is observed two times more frequently in women than in men and usually occurs in middle age [ 4 ]. Transmission can occur, usually by inhalation. However, it can also occur after root canal treatments are applied to the teeth adjacent to the maxillary sinus [ 5 ]. FB is commonly seen in the paranasal sinuses, and its presence is extremely rare in the nasal cavity [ 6 ]. The diagnostic process is vital and dynamic that requires efficient communication and collaboration. The proper investigation is an essential step in the diagnosis, which usually requires imaging; although, imaging alone is not sufficient [ 7 ]. FB displays radiographically as a radiopaque image, which resembles a foreign body [ 8 ]. Definitive diagnosis is obtained only after histopathological examination [ 9 ]. In this case, a rare FB in the vicinity of the nasal cavity has been diagnosed. A microbiological examination supported by radiographic and histopathological results indicated that the FB is due to *Aspergillus niger*.

## Case report

**Figure 1:**
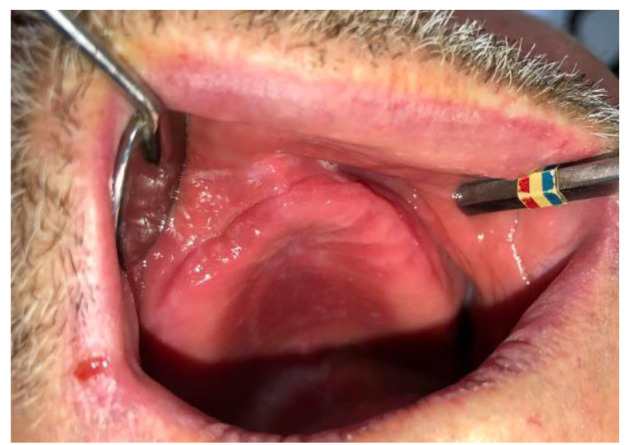
Folded tissue and pus flow are observed on the edentulous crest

**Figure 2:**
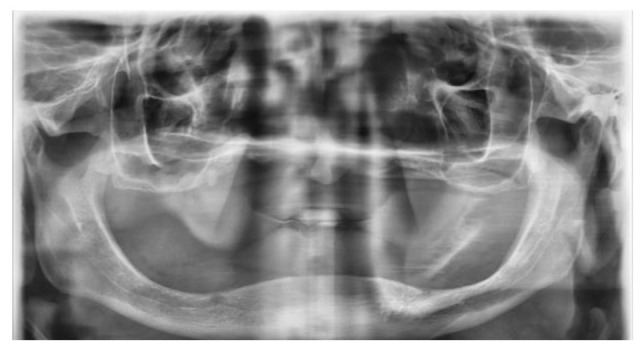
Orthopantogragh of the edentulous maxilla and mandible

A 73-year-old male patient came to our clinic complaining of total missing teeth. When the intraoral examination was performed, an erythematous tissue in the form of epulis and a slight pus flow was found in the edentulous alveolar crest region of the right upper central and lateral tooth (Figure 1 and 2). The remaining root thought to belong to the right upper lateral tooth was observed on the panoramic radiograph. Due to long-term edentulism, the height of 

the alveolar crest was quite low. The patient was advised not to use any dental prosthesis for a week and pay attention to oral hygiene, and then he was called for an observing session. 

**Figure 3:**
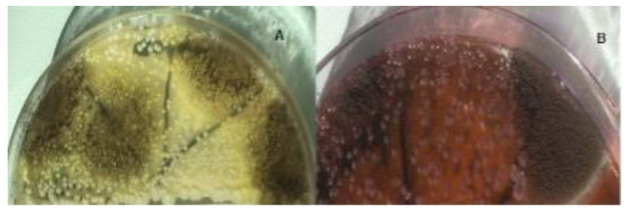
Black colonies with a granular appearance on potato dextrose agar (A) and eosine methylin blue agar (B)

**Figure 4:**
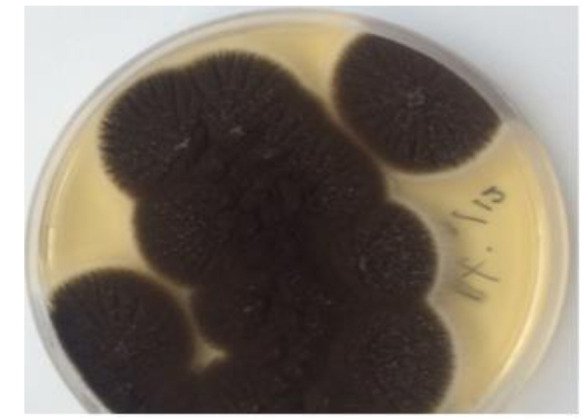
Growth of black colonies with conidial production after 48 hour’s subculture onto potato dextrose agar

After coming to the session, a swab sample was taken from the tissue folds, where there is minimal pus flow. The swab sample was placed in a tube containing 3 mL of 0.1% buffered peptone water and sent to the laboratory for microbiological examination. The sample was then vortexed for 30 sec, and suspension from 0.1 ml dilution was inoculated onto Potato Dextrose Agar (PDA)(Merck, KGaA 64271, Darmstadt, Germany) and eosin methylene blue agar (Merck, KGaA 64271, Darmstadt, Germany), and it was then incubated at 37°C. An apparent growth of black colonies with a granular appearance was obtained after 3 days on both media (Figure 3). Subculture of initial growth was performed on PDA and revealed fungal colonies that were initially white and turned black with the conidial production after 48 h of incubation (Figure 4). Microscopic examination of the colonies revealed smooth and colorless conidiophores. A closer examination showed a globose conidial head, which was dark brown in color divided into a number of columns and covered with dark or dark brown spores (Figure 3). 

Confirmation of the species level of the isolate that was identified with classical methods was confirmed with MALDI-TOF by İzmir Atatürk Training and Research Hospital, Medical Microbiology Laboratory, Medical Mycology Unit. After unexpected results of the microbiological examination, cone beam computed tomography was taken from the relevant region. In the examination of the sections created from dental volumetric tomography performed for the maxilla with a 6×4 cm imaging area and a section thickness of 0.085 mm, a radix of 5×2.4×2.1 mm was observed in the upper right canine tooth. There was a distinct resorptive appearance in the alveolar bone in the imaged area and a radiolucent lesion measuring 10×6.1×9.4 mm in the widest part of the upper right center of the tooth. The lesion was found to be associated with the floor of the nasal cavity and appears to be responsible for the mucosal thickening in the relevant area. 

**Figure 5:**
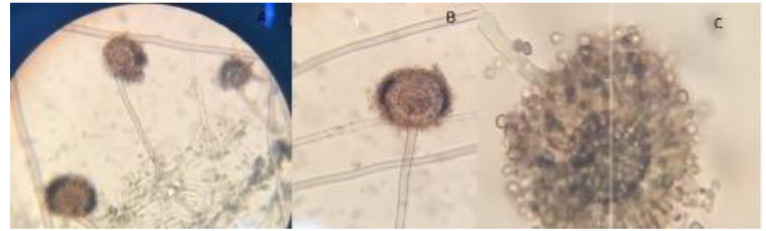
Microscopic examination of Aspergillus niger colonies reveals conidiophores and conidial head (A and B) and conidial head structure divided into a number of columns (C)

Within this lesion, a radiopacity of 2.1×3.4×2.3 mm that was irregularly shaped and was more radiopaque than bone was observed. Mucosal thickening is observed in the right maxillary sinus as far as it is included more peripherally in the image (Figure 5). This described appearance is consistent with the characteristic of FB due to *Aspergilla* species [ 10 ]. The patient was referred to an otolaryngologist for systemic involvement investigation. Blood tests and chest X-ray were requested, and the final results revealed no systemic spread as well as no other chronic disease in the medical history of the patient. The patient, who was diagnosed with *Aspergillus niger*, was brought to the general operating room following routine pre-operative preparations. He was placed under general anesthesia with a nasal tube. A tampon for the throat was placed, and the mouth was irrigated with povidone-iodine solution (BATTICON^®^, 10%; Adeka, Samsun, Turkey) + Saline serum solution. For intraoperative hemostasis and postoperative analgesia, regional and infiltrative anesthesia with a total of 6 cc adrenaline-containing local anesthesia was carried out for the right side of the maxilla, which is the operating area of the patient. 

**Figure 6:**
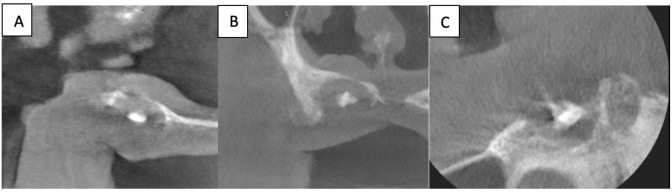
Sagittal (A), coronal (B) and axial (C) sections show a metallic radiopacity consistent with the surrounding fungus ball. Note that is there is a perforation at the floor of the nasal cavity and destruction at the buccal crest

An incision was made under the mucogingival junction with the number 15 scalpel. The full-thickness flap was removed, the granulated tissue described was excised, and the adjacent radix was removed (Figure 6). This area was irrigated with saline and bleeding control was achieved. After the flaps were sutured mainly with 4-0 polyglactin 910 (Vicryl), the surgery was completed by removing the packaging from the throat, and the patient was extubated. Following the awakening, the patient was referred to post-operation room with appropriate recommendations and post-operative order. The patient was prescribed 1000 gr amoxicillin oral tablets for prophylactic purposes. One week later, after the sutures were removed, he was referred for a total denture. Two months after, he started using her prostheses, and he was called for an observing session. He had no complaints, and his overall oral health improved. Moreover, the patient stated that his inhaling comfort was also enhanced. Pathological examination revealed the presence of aspergillosis-associated hyphae and eosinophilic body cysts in the active chronic inflammatory connective tissue. All these results are a strong confirmation of our diagnosis.

**Figure 7:**
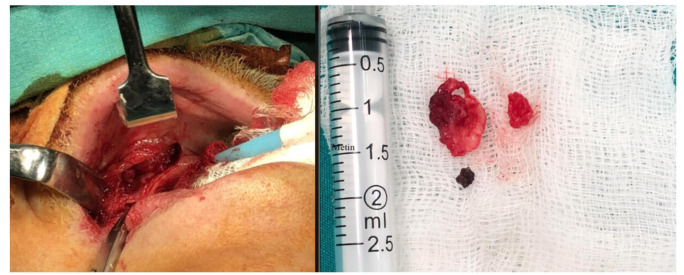
The operation area and the excised tissues, which are predicted to be fungus ball, are observed

## Discussion

Lesions caused by a FB must have the following five characteristics. First, it should be of a non-invasive nature that does not spread to the surrounding tissue. Second, it should have a mucopurulent, clay-like content. Third, there should be opacification within the lesion, with or without calcification. Fourth, there should be a mass of hyphae separated from the adjacent mucosa, and fifth, only allergic mucin, granuloma, or elevated levels of eosinophils should be accompanied [ 11 ]. The results achieved in our case correspond to all these criteria. FBs are generally observed unilaterally with an elevated rate of 94% [ 12 ]. They have been reported as entities that are mostly observed in the paranasal sinuses, and very few of them are observed in the nasal cavity [ 13 ]. In our case, it is believed to have developed in the maxillary alveolar crest with an iatrogenic etiology and merged with the nasal cavity. That is why it can be defined as a rare case. When clinically evaluated, aspergilloma is often overlooked because it is asymptomatic unless the fungal contamination is very long-term [ 14 ]. This could be the reason that our patient did not have any complaints other than missing teeth when applying to our clinic. Many hypotheses have been put forward regarding the etiology of FBs in the nasal cavity. Factors, such as previous dental procedures applied to neighboring teeth, poor oral hygiene, and mucosal trauma, are the ones that are compatible with our case. The pathogenetic mechanism of FBs is explained as follows. An ostial blockage promotes the growth of spores in an anaerobic environment. Fungus salts accumulate more and turned to calcified, hard content. Exogenous and endogenous nidus has been shown to cause this concretion formation [ 15 - 17 ]. 

In our case, we believe that a past root canal treatment resulted in an exogenous nidus. We concluded that the FB formation in the alveolar crest was caused by the previous root canal treatment material that overflowed apically after the treatments that were applied to the teeth in the treated region. This assumption was strengthened after questioning the patient dental history. Previous case reports are also reinforcing this possibility [ 18 ]. During root canal treatment applied to the teeth adjacent to the sinuses, the leakage of the root canal filling material may cause localized inflammation in the sinus mucosa and disrupt mucociliary transport [ 19 ]. 

In fact, foreign material images similar to extruded root canal filling material have been reported in some cases of sinonasal aspergillosis [ 20 ]. In addition, zinc oxide, found in most canal-filling materials, was believed for many years to stimulate the growth of Aspergillosis; however, this belief is no longer supported [ 19 - 21 ]. The chemical reaction between calcium in the patient tissue and oxalic acid, one of the metabolites of the fungus, results in the production of calcium oxalate crystals. *Aspergillus niger* was reported as the fungus with the highest production of these oxalate crystals [ 22 - 24 ]. In this article, *Aspergillus niger* was detected as an etiological fungus, but these crystals were not found in the pathological examination. Treatment of FBs may include endoscopic sinus surgery and depredation [ 25 ]. No case of recurrence of FBs in the nasal cavity has been reported. In our case, the opening to the maxillary alveolar crest provided sufficient intraoral debridement.

## Conclusion

FB is commonly present in the paranasal sinuses; however, it is exceedingly rare in the nasal cavity. Accordingly, proper investigation and diagnosis is a vital and dynamic process that requires effective communication and cooperation. This paper presented the first case of a rare FB in the vicinity of the nasal cavity of a 73-year-old male patient.

This report highlighted a case of fungal infection that is so rare and requires accurate observation. It also featured the importance of meticulous microbiological and histopathological studies combined with imaging, thereby leading to a definitive diagnosis.

## Acknowledgments

Not applicable.

## Authors’ contribution

Ş.E and S.E.K. contributed to the study conception and design. A.B.A. and E.N.K. designed the outline and coordinated the writing of the paper. All authors wrote the original manuscript and assisted in editing. C.G.K performed the surgery.

## Conflicts of interest

The authors declare no conflicts of interest.

## Financial disclosure

This research received no specific grant from any funding agency in the public, commercial, or not-for-profit sectors.

## Ethical considerations

Written informed consent was obtained from the patient. This case was encountered during a study approved by the Ethics Committee of Uşak University, Uşak, Turkey, with the decision numbered 231-01.
